# On-chip plasmonic spin-Hall nanograting for simultaneously detecting phase and polarization singularities

**DOI:** 10.1038/s41377-020-0330-z

**Published:** 2020-05-29

**Authors:** Fu Feng, Guangyuan Si, Changjun Min, Xiaocong Yuan, Michael Somekh

**Affiliations:** 1 Nanophotonics Research Center, Shenzhen Key Laboratory of Micro-Scale Optical Information Technology, Shenzhen University, Shenzhen, 518060 China; 2 Melbourne Centre for Nanofabrication, Victorian Node of the Australian National Fabrication Facility, Clayton, VIC Australia; 3 Faculty of Engineering, University of Nottingham, Nottingham, NG7 2RD UK

**Keywords:** Integrated optics, Nanophotonics and plasmonics

## Abstract

Phase and polarization singularities are important degrees of freedom for electromagnetic field manipulation. Detecting these singularities is essential for modern optics, but it is still a challenge, especially in integrated optical systems. In this paper, we propose an on-chip plasmonic spin-Hall nanograting structure that simultaneously detects both the polarization and phase singularities of the incident cylindrical vortex vector beam (CVVB). The nanograting is symmetry-breaking with different periods for the upper and lower parts, which enables the unidirectional excitation of the surface plasmon polariton depending on the topological charge of the incident optical vortex beam. Additionally, spin-Hall meta-slits are integrated onto the grating so that the structure has a chiral response for polarization detection. We demonstrate theoretically and experimentally that the designed structure fully discriminates both the topological charges and polarization states of the incident beam simultaneously. The proposed structure has great potential in compact integrated photonic circuits.

## Optical singularities: Detecting the indefinable

A device with specially designed nano-sized slits simultaneously detects two indefinable properties in a twisting beam of light. The device, designed by Changjun Min, Xiaocong Yuan, Michael Somekh of China’s Shenzhen University and colleagues, detects phase and polarization singularities in a cylindrical vector vortex beam, with applications in information processing and communications. Optical singularities are regions within light waves where parameters like phase and polarization cannot be defined. Scientists need an easy and reliable way to detect them so they can be manipulated for a variety of applications. In Feng *et al.’s* device, a cylindrical vortex beam shines on specially designed nano-gratings, exciting a wave called a surface plasmon polariton. This wave’s properties depend on the number of twists and degree of spin in the input beam, enabling detection of its singularities. The device is promising for photonic integrated circuits.

## Introduction

Optical singularities are key elements in modern optics and have been widely researched. In particular, phase and polarization singularities have been manipulated in various applications, such as imaging and metrology^1^, nonlinear optics^2^, optical tweezers^3^, sensing^4^, quantum information^5,6^, and optical communication^7,8^.

The phase singularity of an optical field was first theoretically and experimentally demonstrated in the 1970s^9^. Since then, the optical vortex (OV) has been the most widely used optical beam with a phase singularity. The unique properties of the OV, such as a spiral phase wave front, orbital angular momentum (OAM), and donut-shaped intensity distribution, have been used for optical communication in both free space and optical fibres in both classical and quantum regimes to greatly enhance the information transmission ability^6–8,10^. Moreover, owing to the OAM generated by the rotating wave front, OVs can be used in optical tweezers to manipulate nanoparticles^11^. The unique spatial intensity distribution, especially in focusing, also has advantages in imaging and lithography^12^.

The cylindrical vector beam (CVB) is the most commonly used optical beam with a polarization singularity. Two typical CVBs are radially and azimuthally polarized beams in which the electric field oscillates along the radial and azimuthal directions, respectively^13^. Because of their polarization singularity and vector nature, such beams can be used, for example, to generate subwavelength focus^14^ and optical needles^15^. In addition to the solo phase or polarization singularity, an optical beam can have two singularities simultaneously. Such a beam is called a cylindrical vortex vector beam (CVVB)^13^, which provides a higher degree of freedom for modulation and manipulation in optical communication, optical trapping, and microscopy^15–17^.

To use the advantages of a CVVB, it is essential to have an easy and reliable way to detect both phase and polarization singularities simultaneously. It has been proven that a CVVB can be treated as a sum of a left-circularly polarized (LCP) and a right-circularly polarized (RCP) OV beam with different topological charges^18^. The Jones matrix of a CVVB can be expressed as

1 $$J_{l,m} = e^{il\varphi }\left( {\begin{array}{*{20}{c}} {\cos \left( {m\varphi + \varphi _0} \right)} \\ {\sin \left( {m\varphi + \varphi _0} \right)} \end{array}} \right) = \frac{1}{2}e^{i\left( {\left( {l + m} \right)\varphi + \varphi _0} \right)}\left( {\begin{array}{*{20}{c}} 1 \\ { - i} \end{array}} \right) + \frac{1}{2}e^{ - i\left( {\left( {m - l} \right)\varphi + \varphi _0} \right)}(\begin{array}{*{20}{c}} 1 \\ i \end{array})$$

where *φ* is the azimuthal angle, *φ*_0_ is the initial phase angle, *l* is the topological charge value, and m is the polarization order. A CVVB with topological charge *l* and polarization singularity *m* can be expressed as an incoherent sum of an LCP OV beam with topological charge *l* + *m* and an RCP OV beam with topological charge *l* – *m*. In theory, both singularities can be detected simultaneously if one can detect the topological charge and photon spin at the same time. Several methods have been proposed to detect the topological charge of the OAM in recent years, including holography^19,20^, meta-surfaces^21,22^, optical transformation^23–28^, and photonic circuits^29^. However, these methods have drawbacks including the need to align the beam precisely with the structure, the need for complex detection processes, such as near-field microscopy, and the low diffraction efficiencies of some elements. These drawbacks strongly limit their applications in new optical systems with optical fibres or integrated on-chip devices.

In this paper, we propose and demonstrate an on-chip device that can simultaneously detect both phase and polarization singularities of a CVVB using a designed plasmonic spin-Hall nanograting. First, a double grating was designed on a metal surface such that input OV beams with different topological charges can be coupled to a surface plasmon polariton (SPP) wave propagating in different directions depending on the topological charge. Then, the SPP wave is coupled to the far field by two additional coupling gratings placed on the left and right sides to facilitate detection. As the topological charge of the incident OV beam increases, the excited SPP propagates to a higher angle on the metal surface and thus couples out to the far field at a different position relative to the coupling gratings. We can calculate the corresponding topological charge of the incident OV beam by measuring the propagation angle of the generated SPP. However, such a structure cannot detect the spin of the input beam. To solve this problem, a spin-Hall meta-slit has been integrated with the double grating to generate a chiral-response structure, which guides the generated SPP wave to the left or right side depending on the spin of the input beam. Altogether, we demonstrate that the structure generates an SPP wave that propagates to the four quadrants on a metal surface under LCP/RCP light with positive/negative topological charge illumination. As mentioned previously, both the topological charge value and the spin state can be determined from the propagation angle of the SPP wave. Thus, the structure is capable of fully discriminating the phase and polarization singularities of the input beam simultaneously.

## Results

**Fig. 1:**
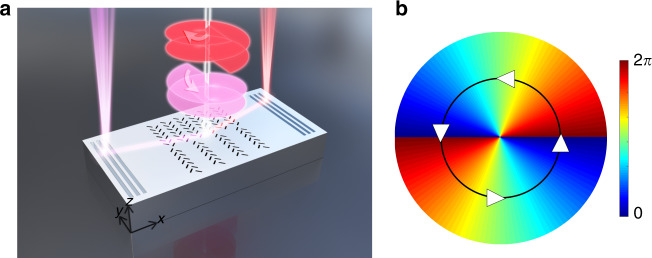
**a** Schematic of the designed structure under illumination of two OAM beams with different polarization states and different topological charges. **b** Spatial phase distribution of an OAM beam with topological charge *l* = 2. The white arrows represent the spatial distribution of the azimuthal k-vector carried by the OAM beam

The designed structure is schematically presented in Fig. 1a. The centre structure is composed of two gratings with different periods *Λ*_*1*_ and *Λ*_*2*_ for the upper and lower parts, respectively. The grating lines are replaced by spin-Hall meta-slits composed of pairs of nanoslits oriented at ±45° and $\mp 45$° for the upper and lower parts, respectively. Such nanoslits give rise to the chiral response of the structure. As the orientations of the upper and lower parts are reversed, the upper and lower parts have an inverted chiral response^17^.

**Fig. 2:**
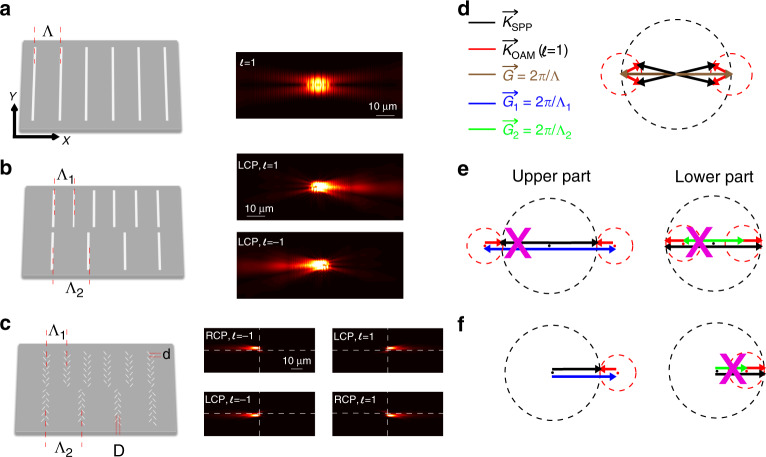
**a**–**c** Left: schematic of a homogeneous nanograting, a double grating, and a spin-Hall meta-slit integrated double grating for the up, middle, and down parts, respectively; right: FDTD simulation results for |E_z_| generated in the left structure under illumination of an LCP OAM beam with topological charge *l* = 1. **d**–**f** Schematic of the k-vector coupling in the left three grating structures with an *l* = 1 LCP OAM incident beam. The dashed black/red circles represent the possible directions of ${\mathop{K}\limits^{\rightharpoonup}\!}_{{{SPP}}}$ and ${\mathop{K}\limits^{\rightharpoonup}\!}_{{{OAM}}}$, respectively; the radius of the circle represents the values of $\left| {{\mathop{K}\limits^{\rightharpoonup}\!}_{{{SPP}}}} \right|$ and $\left| {{\mathop{K}\limits^{\rightharpoonup}\!}_{{{OAM}}}} \right|$; the black/red arrows represent ${\mathop{K}\limits^{\rightharpoonup}\!}_{{{SPP}}}$ and ${\mathop{K}\limits^{\rightharpoonup}\!}_{{{OAM}}}$, respectively; the grey arrow represents ${\mathop{G}\limits^{\rightharpoonup}\!}_0$ introduced by the homogenous grating, as shown in (**a**), and the blue/green arrows represent ${\mathop{G}\limits^{\rightharpoonup}\!}_{1}$ and ${\mathop{G}\limits^{\rightharpoonup}\!}_{2}$ introduced by the upper and lower parts of the asymmetric grating, respectively, as shown in (**b**). For the upper/lower part of the composite grating, only ${\mathop{G}\limits^{\rightharpoonup}\!}_{1}/{\mathop{G}\limits^{\rightharpoonup}\!}_{2}$ is involved in the k-vector coupling process. The violet cross illustrates that the SPP is inhibited

To introduce the design principle of our structure, we first analyse the interaction between a normal grating with a period *Λ* and an incident OAM beam with topological charge *l*. As illustrated in Fig. 2a, particular SPP waves can be excited in two symmetric directions according to k-vector matching. When an OAM beam is sent to the grating, the SPP is launched when the k-vector matching condition

2 $${\mathop{K}\limits^{\rightharpoonup}\!}_{{\mathrm{SPP}}} = \mathop{G}\limits^{\rightharpoonup} + {\mathop{K}\limits^{\rightharpoonup}\!}_{{\mathrm{OAM}}}$$

is fulfilled, where ${\mathop{K}\limits^{\rightharpoonup}\!}_{{\mathrm{SPP}}}$ is the wave vector of the generated SPP wave, $\mathop{G}\limits^{\rightharpoonup} = 2\pi /{\mathrm{\Lambda }}$ is the reciprocal vector of the grating, and ${\mathop{K}\limits^{\rightharpoonup}\!}_{{\mathrm{OAM}}}$ denotes the azimuthal component of the incident OAM beam (as indicated in Fig. 1b). Note that the OAM beam is incident normally to simplify the model. In this case, the only azimuthal wave vector can be expressed as^29–31^

3 $$\left| {{\mathop{K}\limits^{\rightharpoonup}\!}_{{\mathrm{OAM}}}} \right| = \frac{{l\pi }}{{2r}}$$

where *l* represents the topological charge and *r* is the effective radius of the OAM beam. Note that the effective radius of the OAM beam changes the value of the in-plane vector $\left| {{\mathop{K}\limits^{\rightharpoonup}\!}_{{\mathrm{OAM}}}} \right|$_._ It is important that during the experiment, the whole back aperture of the objective is filled by the incident beam so that the size of the focus spot and the $\left| {{\mathop{K}\limits^{\rightharpoonup}\!}_{{\mathrm{OAM}}}} \right|$ value can be well defined.

The k-vector matching process is shown in Fig. 2d. The period of the grating *Λ* equals the effective wavelength of the generated SPP wave. The radius of each circle represents the modulus of the wave vector and the reciprocal vector of the grating. The SPP is generated with an angle *θ* that depends on the direction of ${\mathop{K}\limits^{\rightharpoonup}\!}_{{\mathrm{SPP}}}$ and the topological charge of the incident beam. The angle *θ* can be expressed as

4 $$\theta = 2 \ast arcsin\left( {\frac{{\left| {{\mathop{K}\limits^{\rightharpoonup}\!}_{{\mathrm{OAM}}}} \right|}}{{2 \ast \left| {\mathop{G}\limits^{\rightharpoonup}} \right|}}} \right)$$

One can deduce the topological charge of the incident OV beam by measuring the angle *θ* of the generated SPP. However, this method cannot deduce the sign of the topological charge because the OV beam with topological charge ± *l* generates SPP waves that propagate to identical angles, as shown in Fig. 2a. To identify the sign of the topological charge of the incident OV beam, we analyse the spatial distribution of the azimuthal k-vector of the incident OAM beam. Figure 1b is a schematic of the phase distribution of an OAM beam with topological charge *l* = 2. The black line and white arrow show the spatial distribution of the azimuthal k-vector (phase gradient), where the azimuthal k-vectors point towards the 1st, 2nd, 3rd, and 4th quadrants at different positions of the OAM beam. By breaking the symmetry of the grating, it is thus possible to couple the OAM beam into SPP wave propagating towards one side, which gives rise to the possibility of distinguishing the sign of the topological charge.

Here, we present a symmetry-breaking structure presented in Fig. 2b that can distinguish the sign of the topological charge. The grating is composed of two gratings with different periods *Λ*_*1*_ and *Λ*_*2*_ on the upper and lower parts, respectively. Under the illumination of an OAM beam, when the horizontal centre line of the beam is superposed with the centre line of the gratings, SPP generation occurs. Figure 2e illustrates the k-vector coupling of the structure. The k-vector matching condition is expressed as ${\mathop{K}\limits^{\rightharpoonup}\!}_{{\mathrm{SPP}}} = {\mathop{G}\limits^{\rightharpoonup}\!}_{1,2} + {\mathop{K}\limits^{\rightharpoonup}\!}_{{\mathrm{OAM}}}$, where 1 and 2 denote the upper and lower parts of the grating, respectively. The periods of the upper and lower gratings are designed such that for an OAM beam with topological charge *l* = 1, the three vectors ${\mathop{K}\limits^{\rightharpoonup}\!}_{{\mathrm{SPP}}}$, ${\mathop{K}\limits^{\rightharpoonup}\!}_{{\mathrm{OAM1}}}$, and $\mathop{G}\limits^{\rightharpoonup}$ are collinear and satisfy the equation

5 $$\left| {{\mathop{K}\limits^{\rightharpoonup}\!}_{{\mathrm{SPP}}}} \right| = \left| {{\mathop{G}\limits^{\rightharpoonup}\!}_{1}} \right| + \left| {{\mathop{K}\limits^{\rightharpoonup}\!}_{{\mathrm{OAM1}}}} \right| = \left| {{\mathop{G}\limits^{\rightharpoonup}\!}_{2}} \right| - \left| {{\mathop{K}\limits^{\rightharpoonup}\!}_{{\mathrm{OAM1}}}} \right|$$

The values of *Λ*_*1*_ and *Λ*_*2*_ are thus calculated to be 550 nm and 660 nm, respectively, from Eq. (5).

As shown on the left in Fig. 2e and as explained previously, the in-plane k-vector of the OAM beam only points towards the 2nd and 3rd quadrants for the upper part of the grating (the red arrow points only towards the left). The phase-matching condition is fulfilled only when the SPP propagates towards the right. The generated SPP cannot propagate towards the left under this condition (as illustrated by the violet cross in the figure). For the lower part of the grating, the in-plane k-vector of the OAM beam only points towards the 1st and 4th quadrants. The same phenomena are observed (right side of Fig. 2e) owing to the symmetry-breaking of the grating. Eq. (6) shows that two reciprocal ${\mathop{G}\limits^{\rightharpoonup}\!}_{1,2}$ vectors plus two opposite ${\mathop{K}\limits^{\rightharpoonup}\!}_{{\mathrm{OAM1}}}$ vectors can produce two identical ${\mathop{K}\limits^{\rightharpoonup}\!}_{{\mathrm{SPP}}}$ vectors in the same direction. Under the excitation of an OAM beam with topological charge *l* = 1, the generated SPP beam propagates unidirectionally to the right. In contrast, when the incident beam is changed to an OAM beam with topological charge *l* = –1, similar conditions are fulfilled but in the opposite direction; hence, the generated SPP propagates towards the left in this case. Therefore, with the designed double-grating structure containing two different periods, the incident OAM beam with positive/negative topological charge will unidirectionally couple to an SPP wave towards the right/left. Additionally, Eq. (4) shows that the angle between the generated SPP wave and the X axis is determined by the topological charge. For the moment, the structure is designed such that ${\mathop{K}\limits^{\rightharpoonup}\!}_{{\mathrm{SPP}}}$, ${\mathop{K}\limits^{\rightharpoonup}\!}_{{\mathrm{OAM}}}$, and $\mathop{G}\limits^{\rightharpoonup}$ are collinear when *l* = ±1. The advantage of this design is that higher splitting angles are available for higher order OAM beams. However, for example, as can be observed in Fig. 2c, the SPP generated by the LCP/RCP vortex with *l* = 1 will propagate along the direction. Although the beams are separated spatially, the output spots overlap on the detector, the discrimination of these two modes relies strongly on the single/noise ratio. To overcome this issue, we can adjust the grating period ${\mathop{G}\limits^{\rightharpoonup}\!}_{1}$and ${\mathop{G}\limits^{\rightharpoonup}\!}_{2}$ such that for *l* = ±1, the SPP is generated into a minute angle (increasing $\left| {{\mathop{G}\limits^{\rightharpoonup}\!}_{1}} \right|$ and decreasing $\left| {{\mathop{G}\limits^{\rightharpoonup}\!}_{2}} \right|$ by the same quantity). As a result, the displacements of the output spot for the vortex with *l* = ±1 are no longer zero.

Thus far, we have demonstrated that the phase singularity of an incident OV beam can be characterized using a symmetry-breaking nanograting. To be able to further characterize the polarization singularity of a CVB, the structure needs to have a response to the spin of the light (LCP or RCP). To achieve this, we give each element of the structure a composite structure operating as a spin-Hall meta-slit composed of pairs of plasmonic nanoslits oriented at ± π/4. The length, width, and depth of each nanoslit are 250 nm, 50 nm, and 60 nm, respectively. Because each nanoslit under circularly polarized light illumination can be considered a dipolar source with a relative phase delay of *Δφ* = ±π/4 relative to the incident beam, such a spin-sensitive structure can unidirectionally launch an SPP wave according to the ± sign of the circularly polarized light (± denotes LCP/RCP)^31,32^. By integrating the spin-Hall meta-slit into the above double-grating structure (Fig. 2b), we show that the structure can detect polarization singularities.

**Fig. 3:**
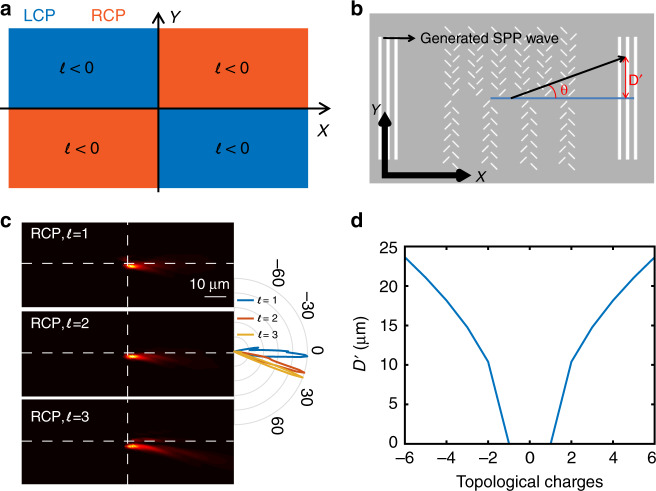
**a** Propagation direction of the generated SPP with an incident OAM beam with different polarizations and topological signs. **b** Schematic of the studied structure. The angle *θ* and displacement *D’* are shown in the figure. **c** Left: FDTD simulation results for |E_z_| when the structure is illuminated by an RCP OAM beam with topological values *l* = 1, 2, and 3, increasing the topological value of the incident beam increases the angle of the launched SPP wave; Right: propagation diagram angle of the generated SPP waves; **d** calculated vertical displacement *D’* between the original and scattered beam spots on the output coupling grating vs. the topological charge

The left side of Fig. 2c is a schematic of how to integrate a spin-Hall meta-slit onto a nanograting. Simply replacing each line on the nanograting with a meta-slit turns the double grating into a chiral-sensitive spin-Hall nanograting structure. The centre of the meta-slit is superposed with the position of the original line so that the period of the meta-slits is guaranteed to be the same as that of the original grating. Each meta-slit is composed of a pair of nanoslits oriented at –45° and +45°. Assuming that the SPP is located at the silver/air interface, the effective wavelength of the SPP on this structure is $\lambda _{{\mathrm{SPP}}} = 2\pi /k_0\sqrt {\varepsilon _{{\mathrm{air}}}\varepsilon _{{\mathrm{ag}}}/\left( {\varepsilon _{{\mathrm{air}}} + \varepsilon _{{\mathrm{ag}}}} \right)} = 610$nm with a 633 nm laser excitation, where *k*_0_ is the wave vector of the incident laser beam and *ε*_air_ and *ε*_ag_ are the dielectric constants of the air and silver, respectively. In this case, the lateral spacing is $D = \lambda _{{\mathrm{SPP}}}/4 = 152.5\,{\mathrm{nm}}$ so that the LCP/RCP light couples to the SPP propagating to the right/left. The vertical spacing *d* should be smaller than *λ* and was chosen to be 200 nm in our study. The two slits in each pair of nanoslits are vertically displaced by 100 nm to save space. The upper and lower parts of the nanograting with periods *Λ*_*1*_ and *Λ*_*2*_, respectively, are replaced by meta-slits with inverted orientations, as shown on the left in Fig. 2c. This design allows the upper/lower part of the spin-Hall nanograting to have an inverted response to incident circularly polarized light. Together with the symmetry-breaking nanograting, the structure can couple the incident circularly polarized OV beam with the SPP as a function of the signs of the topological charge and spin state. Taking LCP light with a positive topological charge as an example, the k-vector coupling permits the incident OV beam to couple with the SPP wave propagating towards the right. Additionally, because the beam is LCP, the upper part of the spin-Hall nanograting allows the excited SPP wave to propagate towards the right, but the k-vector coupling prohibits propagation of the SPP wave towards the left coupled by the lower part of the spin-hall nanograting. At the end, LCP light with a positive topological charge can only couple to the SPP wave that propagates in a single direction, in this case towards the up-right direction (shown in Fig. 2f). The right side of Fig. 2c shows the finite-difference time domain (FDTD) simulation results. The SPP waves launched via different incident beams agree well with the theoretical predictions based on wave vector analysis and the spin-Hall effect. The incident OAM beam with LCP/RCP light and topological charge of ±1 indeed couples with SPP waves that propagate towards the four quadrants. It is thus possible to simultaneously detect the polarization and topological charge of the incident OAM beam. The result is summarized in Fig. 3a.

The exact value of the topological charge can be determined by measuring the angle of the generated SPP wave. An output coupling grating can be introduced with a distance *L* from the centre double-grating structure. The SPP beams will be scattered out at two local spots on the output coupling grating with a displacement *D’* relative to the X axis, which can be calculated as *D’* *=* L*tan *(θ)* (as shown in Fig. 3b). The angle *θ* can be deduced by measuring the displacement *D’*. Figure 3c shows the calculated results for an SPP wave under the illumination of an RCP OAM beam with topological charges *l* = 1, 2, and 3. In all three situations, as predicted, the launched SPP waves propagate towards the 4th quadrant, and the angle between the propagation direction and the X axis increases when the topological charge increases. The exact relation between the vertical displacement *D’* and topological charge can thus be deduced from Eq. (4), and it is shown in Fig. 3d.

To conclude, this structure can be used to identify the value and sign of the topological charge of the incident OAM beam simultaneously by using an output grating to couple the propagating SPP wave to the far field for measurement. The structure can thus fully discriminate the polarization state and the singularity and value of the topological charge.

**Fig. 4:**
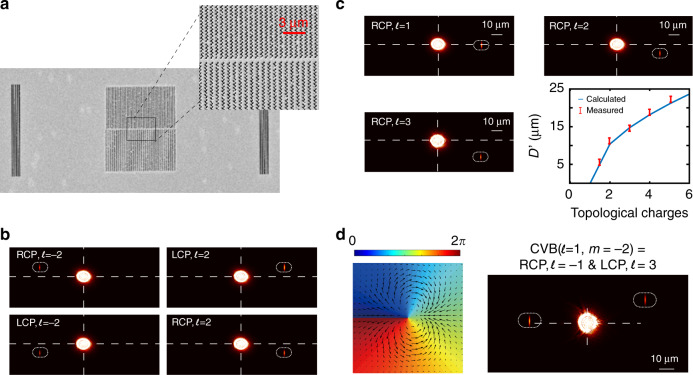
**a** SEM image of the fabricated sample. **b** Image of the sample under the excitation of an RCP OAM beam with topological charges *l* = 1, 2, and 3. **c** Image of the sample under the excitation of an OAM beam with different RCP/LCP beams and topological charges *l* = ±2. **d** Left: spatial phase and polarization distribution of a CVVB with *l* = 1 and *m* = –2; right: optical image of the sample under excitation by this CVVB; the excitation in this case can be considered an RCP OAM beam with *l* = –1 together with an LCP OAM beam with *l* = 3

For the experiment, we fabricated a spin-Hall nanograting on a sample substrate via focused ion beam (FIB) lithography (details are described in the “Methods” section). Figure 4a is the scanning electronic microscopy (SEM) image of the fabricated sample. The nanograting structure was fabricated with the parameters mentioned above. An output coupling grating is added on both sides of the grating at a distance of 40 µm. The period of each output grating equals the effective wavelength of the SPP wave (620 nm). Figure 4b shows the optical microscopy images of the unidirectional coupling of SPPs with OAM incident beams at different polarizations and signs of topological charge (details of the optical setup are described in the “Methods” section). For OAM beams with LCP/positive, RCP/negative, LCP/negative, and RCP/positive topological charges, the SPPs propagate to the 1st, 2nd, 3rd, and 4th quadrants, respectively, as predicted in Fig. 3a. This finding proves that the designed structure indeed simultaneously detects both the polarization and sign of the topological charge of the incident OAM beam.

Figure 4c shows optical microscopy images of the coupling of SPPs with RCP OAM beams with different topological charges. When the incident beam is an RCP OAM with a positive topological charge, coupling occurs only for the SPP waves propagating towards the 4th quadrant. The propagation angle of the launched SPP wave increases as the topological charge increases. The measured vertical displacement between the excitation spot and output coupling spot agrees well with the theoretical predictions, as shown at the bottom right in Fig. 4c. This result shows that the exact value of the topological charge of the incident OAM beam can be determined by measuring the vertical displacement *D’*. It would be difficult practically to discriminate the topological value for an OAM beam with topological value *|l|* > 6. There are essentially two principal reasons: first, the coupling efficiency decreases as *|l|* increases, so that a higher signal-to-noise ratio is required; second, the separation distance decreases. To enhance the performance of the device, one could separate the spin-Hall grating structure with the output coupling grating even farther to have better resolution of the vertical displacement *D’*. Furthermore, because the SPP wave experiences a heavy Ohmic loss during propagation (we estimate less than 1% efficiency for our experiments, which is comparable to other plasmonic devices), other surface waves such as Bloch surface waves (BSWs), which can be propagated on lossless dielectric materials, are attractive candidates for such devices^30^.

Finally, we demonstrate the ability of the structure to detect phase and polarization singularities of a CVVB simultaneously. As mentioned in the previous sections, a structure can detect the phase and polarization singularities of the incident beam simultaneously if it can separate a mixture of LCP/RCP OAM beams with different topological charges. In the last experiment, we generated a CVVB with phase singularity *l* = 1 and polarization singularity *m* = –2 (details are described in the methods section). The beam, in this case, can be considered an incoherent sum of an RCP OAM beam with *l* = –1 and an LCP OAM beam with *l* = 3^13^. The left side of Fig. 4d illustrates the spatial phase and polarization distribution of the in-plane electric field *E*_r_, and the right side of the figure shows the optical microscopy image when such a beam illuminates the sample. Two spots on the output coupling gratings appear simultaneously; the spots on the left and right sides correspond to the couplings of the SPP wave to the RCP OAM beam with *l* = –1 and the LCP OAM beam with *l* = 3^13^, respectively. The phase and polarization of the incident CVVB beam can then be determined to be *l* = 1 and *m* = –2. Thus, the full detection of the phase and polarization singularities of the incident beam is achieved using the proposed spin-Hall nanograting structure. Compared with other devices, our device is alignment-free (the beam and the grating do not need precise spatial matching). Moreover, the detection is far-field detection, which does not involve complex optical systems such as near-field scanning optical microscopes.

## Discussion

In this study, we have theoretically and experimentally demonstrated a plasmonic spin-Hall nanograting that can simultaneously detect the phase and polarization singularities of the incident beam. A symmetry-breaking nanograting structure was designed first to unidirectionally launch the SPP wave according to the sign of the topological charge of the incident wave. The propagation angle of the generated SPP increases with the value of the topological charge. The topological charge value of the incident beam can be accurately determined by placing an output coupling grating on both sides of the nanograting to couple the generated SPP wave to the far field and analysing the far-field optical microscopy image. A spin-Hall structure is then integrated onto the nanograting so that the nanograting can respond to the spin of the incident beam. This combined structure directionally couples the incident OAM beam to different positions depending on the polarization and topological charge of the beam. We finally proved experimentally that the structure detects the polarization singularity and phase singularity of the incident CVB beam simultaneously. This device is very promising for achieving a highly compact photonic integrated circuit. It is also possible to replace the SPP wave with alternative surface waves, such as BSWs, which show great potential in large-scale photonic integrated circuits and would benefit diverse applications such as optical on-chip information processing and optical communications.

## Materials and methods

### Fabrication procedure

A 60-nm-thick silver film was deposited onto a quartz substrate via electron-beam evaporation with a 5-nm germanium adhesion layer. The spin-Hall nanograting structures were fabricated using focused ion beam milling (FEI Helios NanoLab600 DualBeam FIB-SEM system). During focused ion beam milling, a 28-pA beam current was used with an accelerating voltage of 30 kV.

### FDTD simulations

For all the FDTD simulations, the analysis structure was a 60-nm silver (*n* = 0.14 + 4.15i) layer on top of a SiO_2_ (*n* = 1.45) substrate. The wavelength of the incident beam was set to 633 nm. Perfectly matched layers (PMLs) are placed around the entire structure. A global mesh of λ/10 and an additional mesh of λ/20 were applied to the grating area.

### Experimental setup

**Fig. 5:**
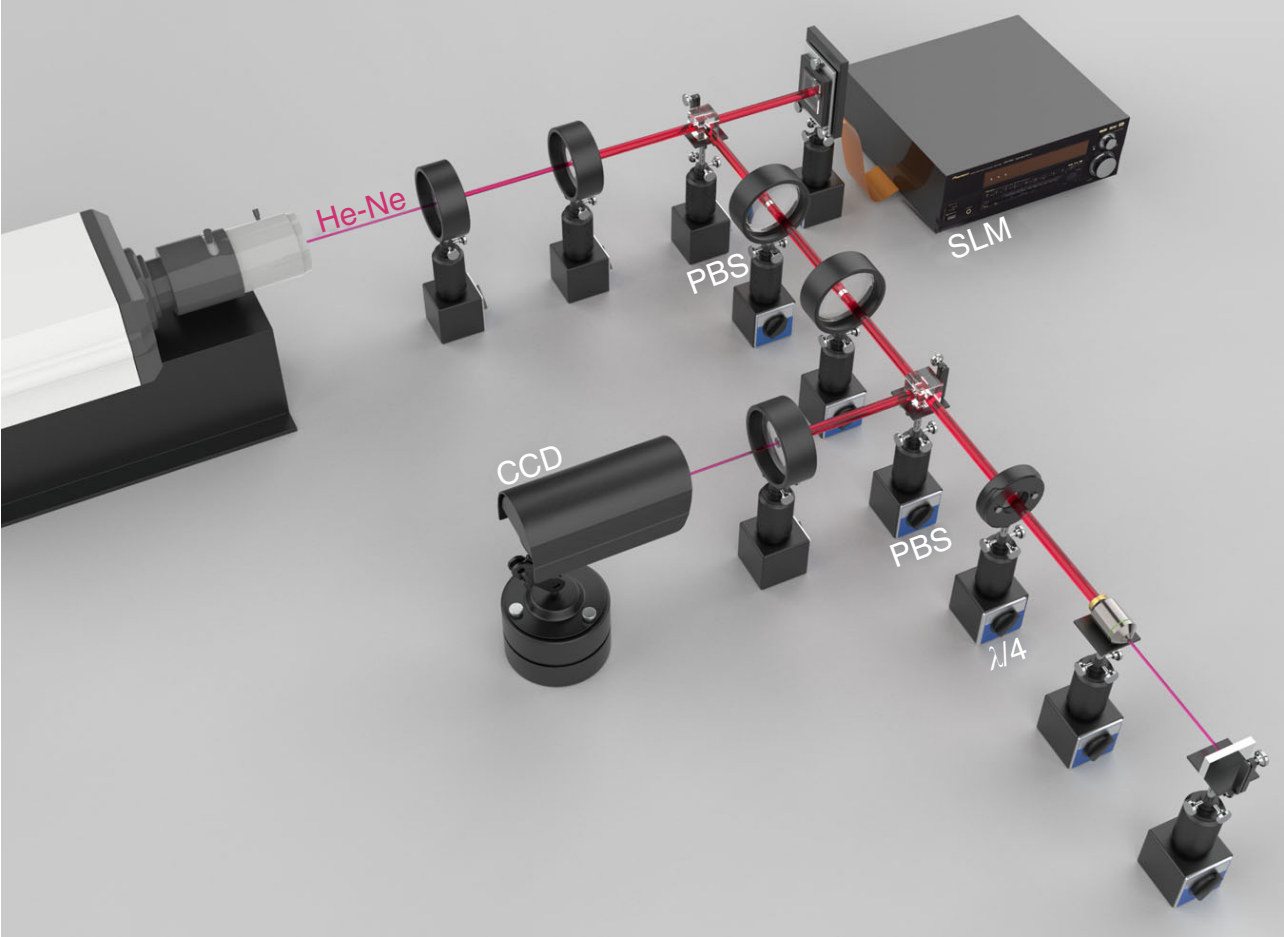
Schematic of the experimental setup

The optical images presented in the paper were recorded with the experimental setup shown in Fig. 5. A He–Ne laser (HRS015B, Thorlabs) with a wavelength of 633 nm was used as the optical source. The laser beam was first expanded with two lenses and passed through a polarized beam splitter (PBS) to cover the entire spatial light modulator (SLM, Meadowlark P1920-0653-HDMI) surface and guarantee the input light polarization. A helical phase pattern was added to the SLM to modulate the input beam to different OAM beams. The beam was focused on the sample by a ×40 objective lens with a numerical aperture of 0.65. A quarter-wave plate was placed in front of the objective lens to change the polarization state of the input beam. The reflected light was focused on a CCD camera (Thorlabs CS505MU) for data recording.

To generate the CVVB, a helical phase pattern corresponding to the phase pattern of an OAM beam with *l* = 1 was loaded on the SLM. The beam then passed through a vortex wave plate with *m* = 2 (Thorlabs WPV10–633). The beam thus carried phase and polarization singularities *l* = 1 and *m* = –2. The beam was then focused by the objective lens onto the sample. The reflected beam was collimated by the same objective lens and focused on the CCD camera for data recording.

## Supplementary Information

Supplementary Information
