# Supplementary material for: On-chip plasmonic spin-Hall nanograting for simultaneously detecting phase and polarization singularities
Source: Light Sci Appl. 2020 May 29;9:95. doi: 10.1038/s41377-020-0330-z (PMC7260171; doi:10.1038/s41377-020-0330-z)
Supplement: Supplementary file 1 — Supplementary Information [file 41377_2020_330_MOESM1_ESM.docx]

**Supplementary Information for**

**On-chip plasmonic spin-Hall nano-grating for simultaneously detecting phase and polarization singularities**

Fu FENG^1^, Guangyuan Si^2^, Changjun Min^1*^, Xiaocong Yuan^1^, Michael Somekh^1,3*^

Affiliation:

1. Nanophotonics Research Center, Shenzhen Key Laboratory of Micro-Scale Optical Information Technology, Shenzhen University, Shenzhen 518060, China
2. Melbourne Centre for Nanofabrication, Victorian Node of the Australian National Fabrication Facility, Clayton, Victoria, Australia

Faculty of Engineering, University of Nottingham, Nottingham NG7 2RD, UK

**Beam size and k-vector coupling process:**

It can be noticed that the beam diameter plays an important role in the k-vector matching process here as it changes the azimuthal component of the incident OAM beam denoted as K_OAM_ in Eqs (2) and (3). It is true that the beam diameter plays an important role in the k-vector matching process here as it changes the azimuthal component of the incident OAM beam denoted as K_OAM_ in Eqs (2) and (3). Fig. S1 is the calculated relation between lateral displacements *D’* and beam radius *r* for OAM beam with different topological value *l* from Eqs (4). It can be seen that a beam radius changes from 1 μm to 1.5 μm there is some ambiguity in unique determination of *l* and *m*. In order to avoid such issue, in practice the beam should be expanded to cover the entire rear aperture of the objective so that the focused spot size can be well defined by the numerical aperture of the objective. Furthermore, a grating with phase gradient in the lateral directions (by changing the aspect ratio of spin-hall meta-slit pairs) may further compensate the inaccuracy introduced by the beam radius.


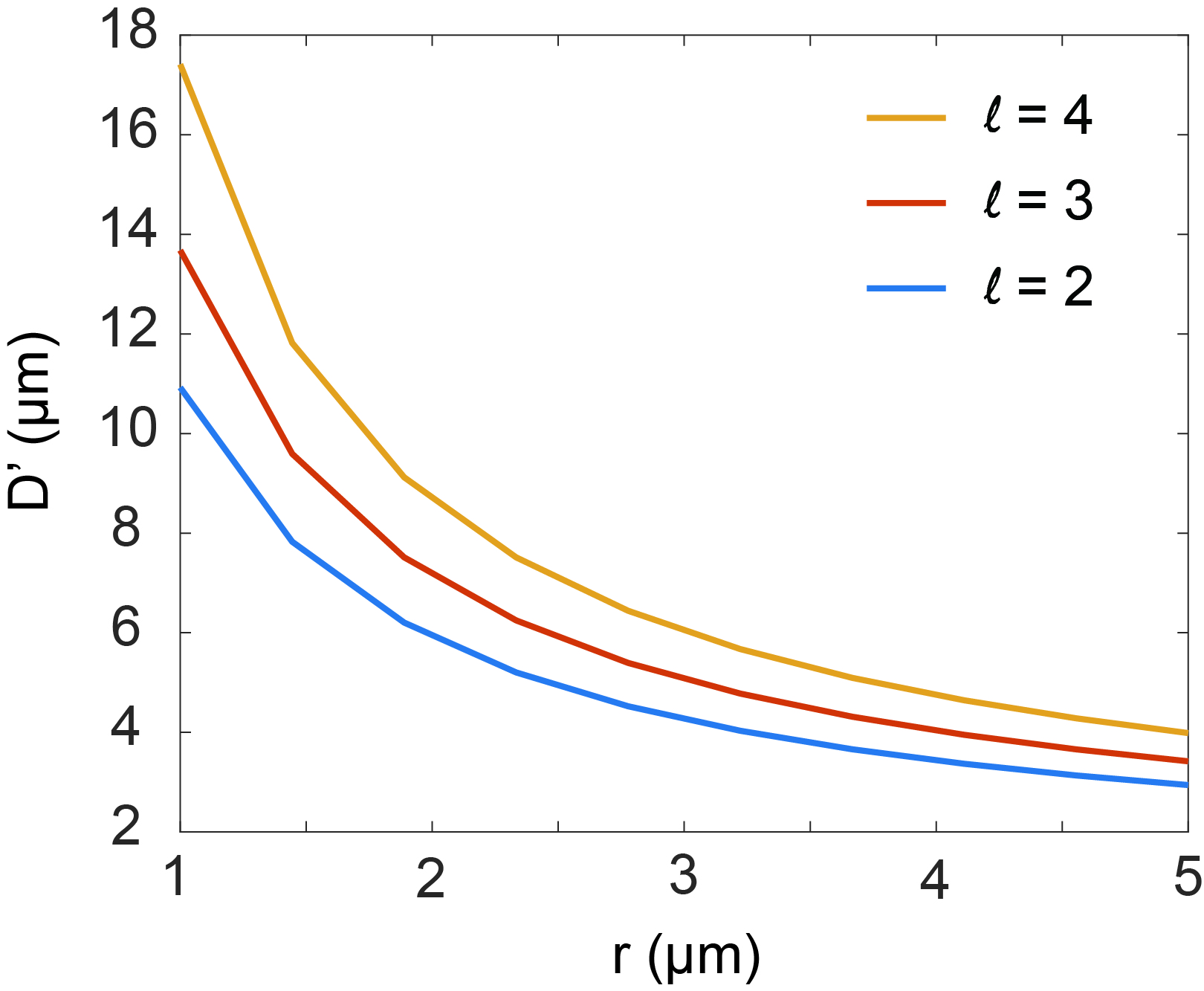


Fig. S1 Relation between lateral displacements *D’* and beam radius for OAM beams with different topological charges.

**Efficiency of the device and Ohmic loss during propagation:**

The efficiency of the device is estimated to be less than 1% by calculating power received by the CCD camera, output power of the laser, loss introduced by the optics in the experimental systems. The efficiency is comparable with other plasmonic devices. There are mainly two kinds of losses in the detecting procedure. First kind is the surface wave generation efficiency (how much incident light can be transmitted into propagative plasmonic modes), this can be improved by using materials with higher optical index contrasts or by incident the beam close to the critical angle (using prism or oil objective) to fulfill the phase match condition. Second loss is the Ohmic loss of SPP during propagation, this can be solved by using alternative surface waves such as BSW wave proposed in the paper also if the beams are in the MIR then the losses are much lower greatly mitigating these effects.

As known [1], the propagation distance of a SPP wave is determined by the substrate material and operation wavelength, it can be calculated by the following equation:

$$L'= \frac{1}{2K^{"}}=\frac{c}{\omega}\left( \frac{\varepsilon_{0}+\varepsilon_{m}^{'}}{\varepsilon_{0}\varepsilon_{m}^{'}} \right)^{\frac{3}{2}}\frac{\left( \varepsilon_{m}^{'} \right)^{2}}{\varepsilon_{m}^{"}}$$

Where $K^{"}$is the imaginary part of the wave vector of generated SPP wave, *c* is the speed of light, ω is the angular momentum of incident beam, *ε_0_* is the dielectric constant of air and $\varepsilon_{m}= \varepsilon_{m}^{'}+ \varepsilon_{m}^{"}$ is the dielectric constant of metal. With our device, the propagation distance *L’* is few tens of micrometer approximately. The relative short propagation distance of SPP is a key limiting factor for our device. As our detection method is to scatter the SPP wave out to the far field by a scattering grating and measure the lateral displacement of the spot. The distance between the coupling structure and the output coupling grating determine the resolution of our device. Fig. S2 is the calculated relative distance of between output spots with respect to distance *L* between the structure and the output coupling grating.


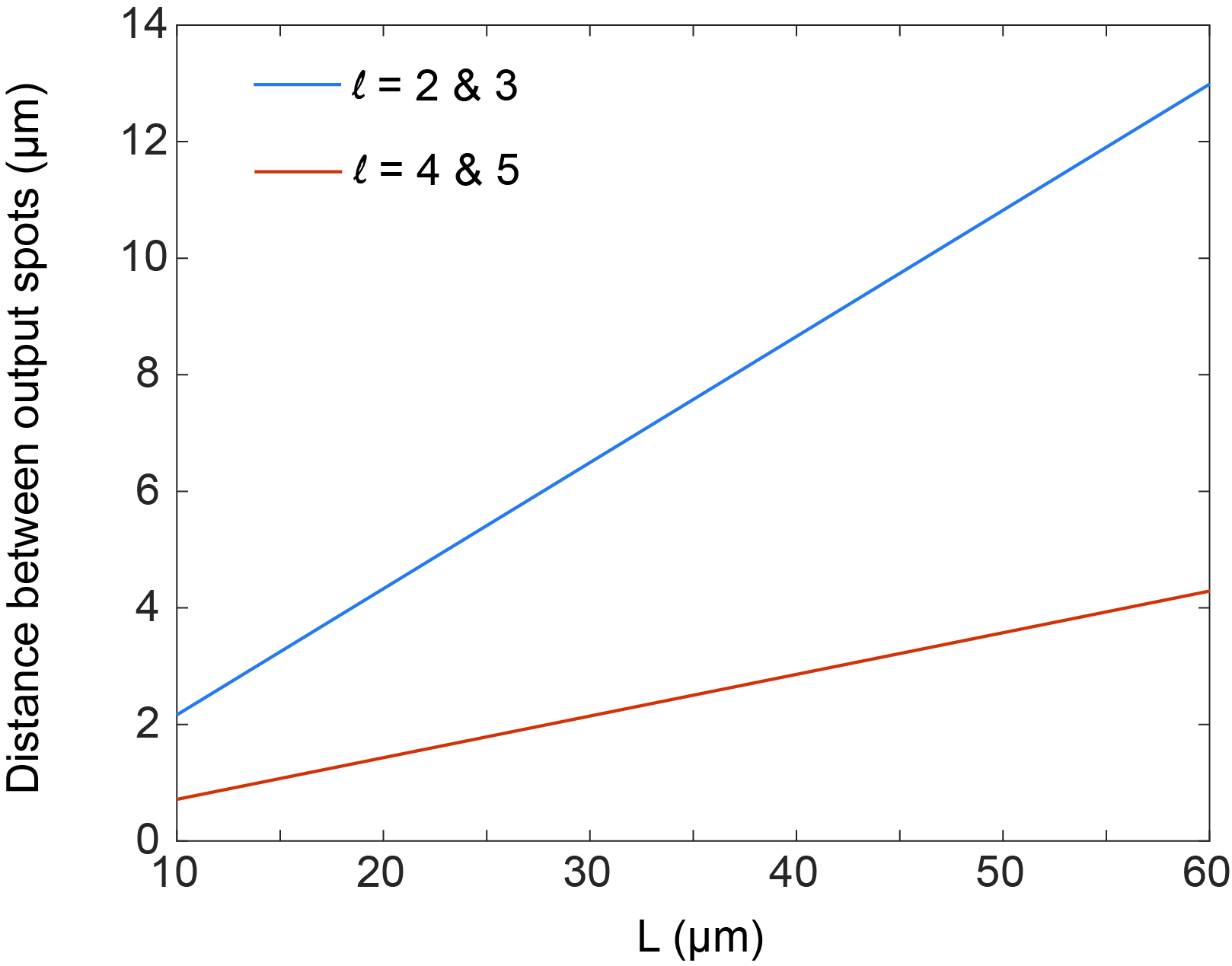


Fig. S2 Relation between *L* and distance between output spots on the output grating

It can be observed that, the distance between output spots generated by OAM beams with different orders increases as when the distance *L* increases. This indicates that further the output grating is placed to the structure, better the resolution will be. Also, for higher order mode, the distance between the output spots are smaller compare to lower order modes, it is thus important to choose a propagation distance *L* which is not too small so that SPP waves generated OAM beams with different orders can be well separated. However, as discussed previously, the SPP experience attenuation during propagation due to Ohmic loss, it is not practical to place the output grating too far away from the structure as single/noise decreases with respect to the distance. A compromise distance *L* need to be achieved to satisfy these two requirements simultaneously. To overcome this issue, a Bloch surface wave (BSW) can be used to replace the plasmonic substrate. As BSW is an alternative surface wave which propagates on top of a multilayer dielectric surface, it does not experience Ohmic loss during propagation, the distance *L* can thus be chosen to be a much bigger value(even to mm scale). The resolution of phase and polarization singularity *l* and m values of a CVVB can be much higher in this case. In the same time, higher order modes can also be well separated after a long propagation distance, the bandwidth of the structure can thus be also improved.

**Detection for pure CVB wave and beam with fractional topological charges.**

If the topological charge of the incident beam is zero. The incident beam becomes a pure CVB beam with polarization singularity m. As indicated by Eqs. 1 in the manuscript, the Jones matrix of a CVVB can be expressed as follows:

$$J_{l,m}=e^{il}\left( \begin{matrix} \cos\left( m+{}_{0} \right) \\ \sin\left( m+{}_{0} \right) \end{matrix} \right)= \frac{1}{2}e^{i\left( \left( l+m \right)+{}_{0} \right)}\left( \begin{matrix} 1 \\ -i \end{matrix} \right)+\frac{1}{2}e^{-i\left( \left( m-l \right)+{}_{0} \right)}(\begin{matrix} 1 \\ i \end{matrix})$$

For the case *l* = 0, the equation can be simplified as:

$$J_{m}=\left( \begin{matrix} \cos\left( m+{}_{0} \right) \\ \sin\left( m+{}_{0} \right) \end{matrix} \right)= \frac{1}{2}e^{i\left( m+{}_{0} \right)}\left( \begin{matrix} 1 \\ -i \end{matrix} \right)+\frac{1}{2}e^{-i\left( m+{}_{0} \right)}(\begin{matrix} 1 \\ i \end{matrix})$$

Which is an incoherent sum of a LCP OAM beam with *l* = *m* and a RCP OAM beam with *l* = -*m*. Thus, the incident beam will couple to SPPs propagating towards two directions accordingly.

If the incident beam CANNOT be expressed by a conventional CVB beam, there might be no directional coupling to SPP in this case.

For a fractional OAM beam, our method is not valid as OAM beam with a fractional charge is not a stable mode and the phase discontinuity will break the k-coupling process and leads to strange output beams. Figure below is a FDTD simulation result of the structure with an OAM beam with *l* = 2.5. It can be observed that the structures can no longer uni-directionlly launch SPP wave in this case.


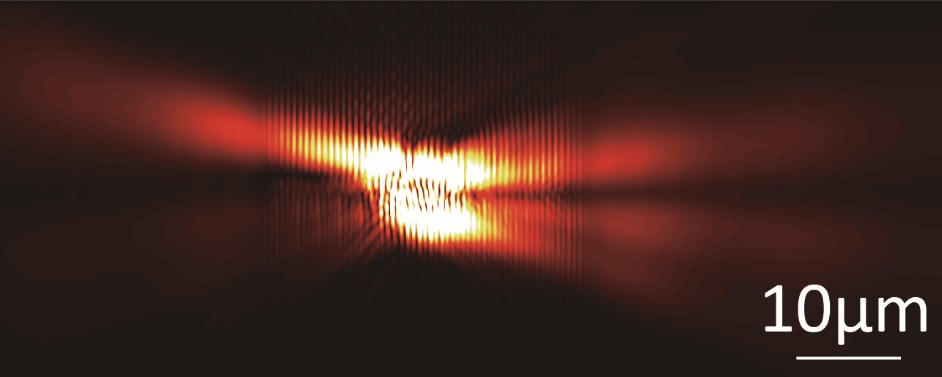


Figure S3 FDTD simulation results for |E_z_| generated under illumination of an OAM beam with *l* = 2.5

**Parameter optimization for the structure**

1. **Thickness of the silver layer and periods of the gratings:**


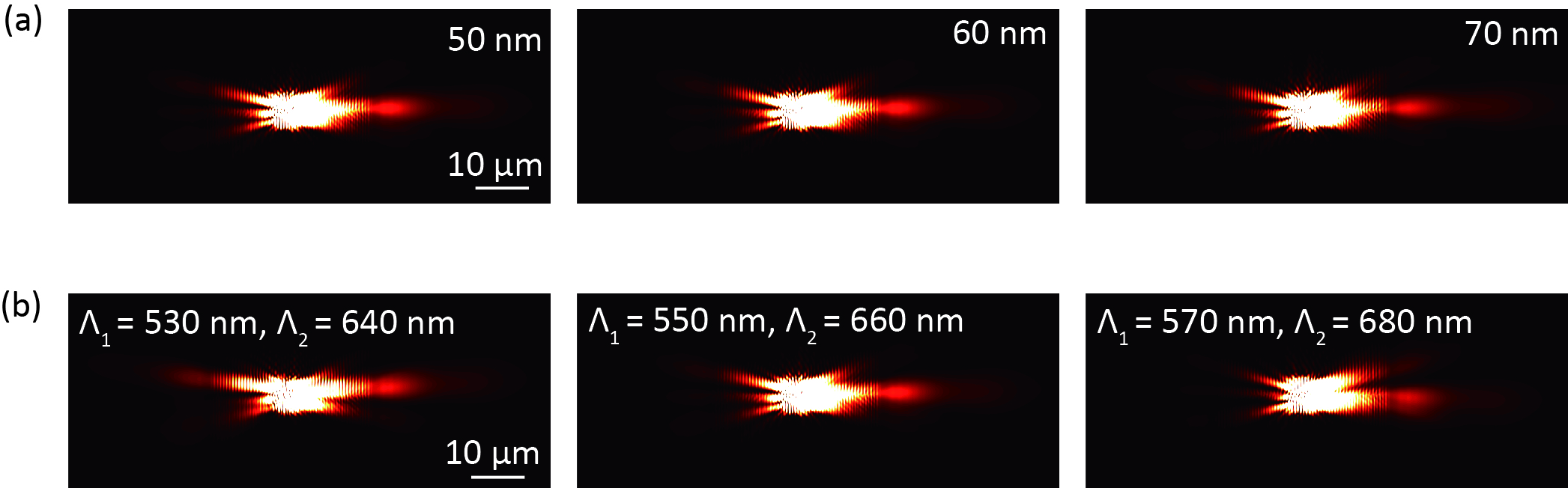


Figure S4 (a) Simulated |Ez| field distribution of composite grating structure with grating period *Λ_1_* = 550n and *Λ_2_* = 660nm for upper and lower part respectively, the structure isunder illumination of an OAM beam with *l* = 1, the thickness of silver on top is 50nm, 60nm, 70nm from left to right respectively; (b) Simulated |Ez| field distribution of composite grating structure with different period *Λ_1_* and *Λ_2_* under illumination of an OAM beam with *l* = 1, the thickness of silver on top is set to be 60nm in this case.

It can be observed from Fig. S4a that the thickness of silver layer does not much change the performance of the structure. This is due to the fact that the thickness we chose (60nm) is much larger than the skin depth of silver (approximately 25nm) at this wavelength, the silver layer can thus be considered as infinite in this case, a slight change of thickness will not change the value of *K_SPP_* so that the coupling process still works. However, from Fig. S4b, when the period of the gratings are changed, the k-vector coupling process breaks rapidly. This can be easily understood with by Eqs (2), when the period of gratings change, the in-plane vector of the gratings $G_{1}$ and $G_{2}$ changes, the k-vector coupling process is no longer valid, thus the incident OAM beam couples to SPP wave into directions not well defined as shown in Fig. S4b. It is very important to choose carefully the periods of the composite gratings according to the laser wavelength, silver thickness in order to have the best coupling.

1. **Parameters of the Spin-Hall meta-slit**


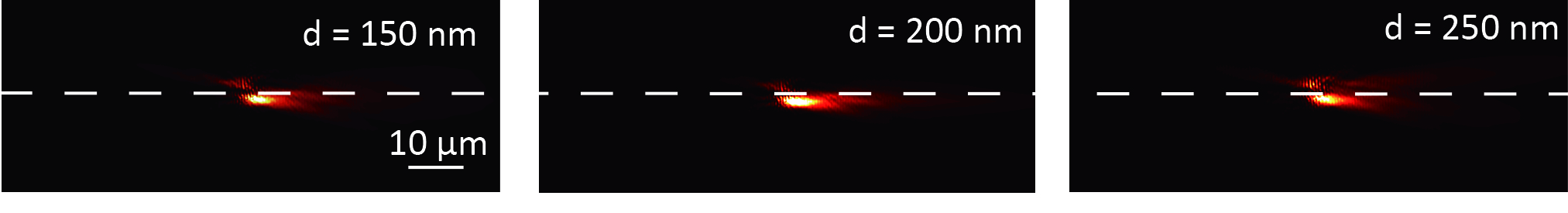


Figure S5 Simulated |E_z_| field distribution of studied structure under illumination of RCP OAM with *l* = 1, the vertical spacing for Spin-Hall meta-slit *d* = 150nm, 200nm, 250nm for the left, middle, right figures respectively

Fig. S5 is simulated |E_z_| field distribution of the composite grating integrated with spin-hall meta-slit with different vertical spacing d under illumination of RCP OAM with *l* = 1. It can be seen that by changing the vertical spacing d for spin-hall meta-slits, the performance of the structure does not change much.


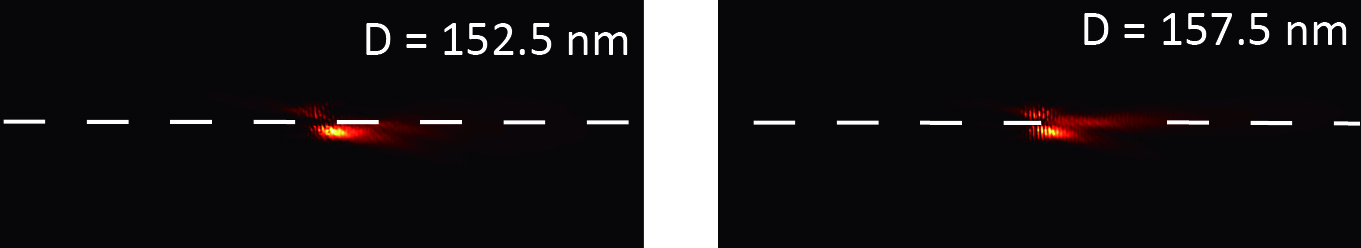


Figure S6 Simulated |E_z_| field distribution of studied structure under illumination of RCP OAM with *l* = 1, the later spacing for Spin-Hall meta-slit *D* = 152.5nm, 157.5nm for the left, right figures respectively

Fig. S6 is the simulated |E_z_| field distribution of the composite grating integrated with Spin-Hall meta-slit with different lateral spacing *D* under illumination of RCP OAM with *l* = 1. It can be observed from the figures that the lateral spacing *D* will affect the performance significantly. When *D* is set to λ_SPP_/4 = 152.5nm, incident beam only couples to the lower part of the grating and SPP propagates towards 4^th^ quadrant. However, if *D* is different from λ_SPP_/4 (157.5nm as shown in Fig. S6 right), the incident beam couples to both upper and lower part of the grating and the generated SPP propagates toward both 1^st^ and 4^th^ quadrants. This can be explained by looking into the details of the working mechanism of the Spin-Hall meta-slit. On the silver surface, each nano-slit can be treated as a dipolar source, when a RCP incident beam is focued on the structure, the SPP wave generated by the nano-slit pairs (one oriented at 45° _­_other at 135°) experiences a phase delay of π/2. If the two nano-slits are positioned with D = λ_spp_/4, an additional phase delay of ±π/2 (+/- signs determined by the propagation direction) will be introduced to be generated SPP waves. The generated SPP waves will interfere constructively (total phase delay π/2 - π/2 = 0) on one side and interfere destructively (total phase delay π/2 + π/2 = π) on the other side (details are explained in ref. 32 of the manuscript). For the simulated result on the right, the Spin-Hall meta-slit does not show response to the polarization of the incident beam, the SPP waves are thus generated for both upper and lower part. It is interesting to notice that in this case, the composite grating still works properly, SPP are all directed to the right side because *l* > 0. It is thus very important to keep the lateral displacement *D* of a meta-slit pair at λ/4 (in our case 152.5 nm).

**Reference:**

[1] Principles of nano-optics Lukas Novotny-Bert Hecht- Cambridge University Press - 2019
